# NEDD9 and BCAR1 Negatively Regulate E-Cadherin Membrane Localization, and Promote E-Cadherin Degradation

**DOI:** 10.1371/journal.pone.0022102

**Published:** 2011-07-12

**Authors:** Nadezhda Tikhmyanova, Erica A. Golemis

**Affiliations:** 1 Developmental Therapeutics, Fox Chase Cancer Center, Philadelphia, Pennsylvania, United States of America; 2 Department of Biochemistry, Drexel University School of Medicine, Philadelphia, Pennsylvania, United States of America; Institut Curie, France

## Abstract

The Cas scaffolding proteins (NEDD9/HEF1/CAS-L, BCAR1/p130Cas, EFSSIN, and HEPL/CASS4) regulate cell migration, division and survival, and are often deregulated in cancer. High BCAR1 expression is linked to poor prognosis in breast cancer patients, while upregulation of NEDD9 contributes to the metastatic behavior of melanoma and glioblastoma cells. Our recent work knocking out the single Drosophila Cas protein, *Dcas*, identified a genetic interaction with E-cadherin. As E-cadherin is often downregulated during epithelial-mesenchymal transition (EMT) prior to metastasis, if such an activity was conserved in mammals it might partially explain how Cas proteins promote aggressive tumor behavior. We here establish that Cas proteins negatively regulate E-cadherin expression in human mammary cells. Cas proteins do not affect E-cadherin transcription, but rather, BCAR1 and NEDD9 signal through SRC to promote E-cadherin removal from the cell membrane and lysosomal degradation. We also find mammary tumors arising in MMTV-polyoma virus T-antigen mice have enhanced junctional E-cadherin in a *Nedd9^−/−^* background. Cumulatively, these results suggest a new role for Cas proteins in cell-cell adhesion signaling in cancer.

## Introduction

The non-catalytic scaffolding proteins of the Cas family control attachment, migration, cell cycle, and cell survival signaling (reviewed in [1], [2], [3]) in mammalian cells. In vertebrates there are 4 family members: BCAR1/p130Cas [4], NEDD9/HEF1/Cas-L [5], EFS/Sin [6] and CASS4/HEPL [7], of which BCAR1 and NEDD9 have been the most intensively studied. A growing number of studies have found that increased expression of Cas proteins contributes to human tumor aggressiveness (reviewed in [3], [8]). BCAR1 overexpression confers invasiveness to cultured cells, and promotes mammary tumorigenesis and lung metastasis in the MMTV-HER2 and other mouse models of cancer [9], [10]. BCAR1 overexpression also correlates with poor prognosis in breast cancer patients [11], [12]. NEDD9 overexpression is frequent in glioblastomas [13], melanomas [14], and some lung cancers [15], and promotes metastasis; upregulation of NEDD9 also promotes oncogenic signaling in the hematopoietic system [16], [17], [18], [19], [20], and supports invasive behavior in breast cancer cell lines [21], while genetic ablation of NEDD9 limits mammary tumor growth in the MMTV-polyomavirus middle T (PyVT) model of tumorigenesis [22], [23].

Tumor invasiveness often requires epithelial-mesenchymal transition (EMT), during which cells lose lateral attachments to their neighbors and become more motile. One of the hallmarks of EMT is downregulation of the cell-cell adhesion protein E-cadherin, resulting in destabilization of the adherens junctions (AJs) that connect cells [24]. Mutations in E-cadherin, and methylation of the E-cadherin promoter are described as common causes of E-cadherin downregulation in human tumors, but are not found in all tumors that have lost E-cadherin expression. Another common mechanism for downregulation of E-cadherin in EMT is transcriptional inhibition based on enhanced action of the transcriptional repressors such as Snail or SLUG (reviewed in [24]). Post-translationally, equilibrium expression of E-cadherin at the plasma membrane is maintained by a regulated balance between exocytosis and endocytosis [25]. Perturbation of this balance can also results in E-cadherin removal from the plasma membrane [25], [26], providing an additional point of control for E-cadherin downregulation in carcinomas.

Some recent results raise the possibility that Cas proteins might influence E-cadherin expression. A 2008 clinical study of E-cadherin and BCAR1 in hepatocellular carcinoma identified a negative correlation between the expression of these two proteins [27], while another work has demonstrated that the environmental pollutant dioxin induces EMT through a pathway involving NEDD9 [28]. The Cas proteins influence the activation of the SRC and FAK kinases [7], [22], [29], [30], and Rho GTPases [31], [32], which contribute to regulation of EMT-linked disassembly of E-cadherin complexes at AJs (discussed in [33]). In a recent study by our group, we found that genetic deletion of the single Cas family member in Drosophila, Dcas, was synthetically lethal with mutations in E-cadherin, and its effectors β-catenin and p120-catenin [34]. In embryos lacking Dcas, E-cadherin levels at lateral cell contacts were significantly reduced during development, although overall intracellular levels of E-cadherin were increased [34]; these results suggested a defect in E-cadherin localization in the absence of DCas caused signaling defects leading to a paradoxical upregulation of E-cadherin. Based on these reports, we investigated Cas protein regulation of E-cadherin in mammals. We have found that NEDD9 and BCAR1 signal through SRC to negatively regulate membrane localization of E-cadherin and its interacting catenins, and in contrast to Drosophila, enhance the lysosomal degradation of E-cadherin pools, leading to a net loss of intracellular E-cadherin. These results suggest a new mechanism by which overexpression of NEDD9 or BCAR1 may contribute to aggressiveness in human tumors.

## Results

### Cas negatively regulates E-cadherin protein expression in human cells

**Figure 1:**
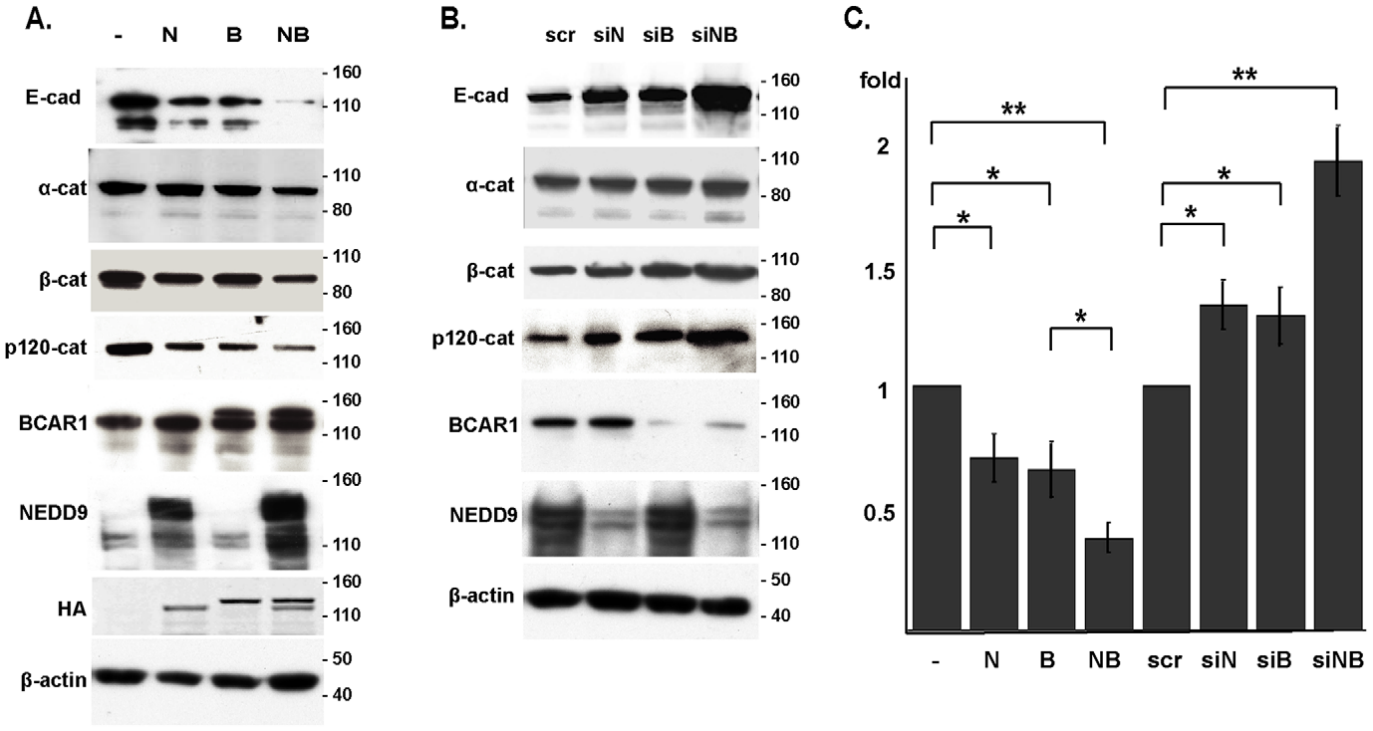
Cas proteins negatively regulate E-cadherin expression in MCF7 cells. **A**. Western analysis of MCF7 cells transfected with plasmids including vector pcDNA-HA (-), pcDNA-HA-NEDD9 (N), pcDNA-HA-BCAR1 (B), or pcDNA-HA-NEDD9 and pcDNA-HA-BCAR1 (NB). **B**. Western analysis of MCF7 transfected with siRNAs including scrambled control (scr), or targeting NEDD9 (siN), BCAR1 (siB) and both (siNB), probed with antibodies indicated. **C**. Graph represents total levels of E-cadherin normalized to β-actin. *, P<0.01, **, P<0.001. Error bars represent SE. Additional siRNA experiments (not shown) were performed with alternative siRNA oligonucleotides targeting NEDD9 and BCAR1: although knockdown was not as efficient, qualitatively similar results were obtained in regard to E-cadherin expression.

The MCF7 breast adenocarcinoma cell line has frequently been used to study function of Cas proteins, and their activity in promoting migration and invasion by these cells is well established [21]. We used breast carcinoma MCF7 cells to overexpress (Figure 1A) or siRNA-deplete (Figure 1B) BCAR1 and NEDD9, individually or in combination, and monitored total expression of E-cadherin and its partner proteins α-, β-, and p120catenin. E-cadherin levels were downregulated in cells overexpressing BCAR1 or NEDD9, and to a greater extent in cells ovexpressing both proteins (Figures 1A, 1C). Reciprocally, and in contrast to our results in Drosophila [34], E-cadherin protein accumulated in cells with either NEDD9 or BCAR1 depleted, and to a higher level in cells with both proteins depleted (Figures 1B, C). E-cadherin associates with α-, β-, and p120catenin at cell junctions, and we had previously identified genetic interactions between DCas and the Drosophila orthologs of these proteins [34]. Extending this analysis, we found that changes in Cas protein expression also negatively regulated the expression of α-, β-, and p120catenin, but to a lesser degree that that seen with E-cadherin (Figures 1A,B).

### Cas proteins induce E-cadherin downregulation from cell junctions and the detergent-insoluble cell fraction

**Figure 2:**
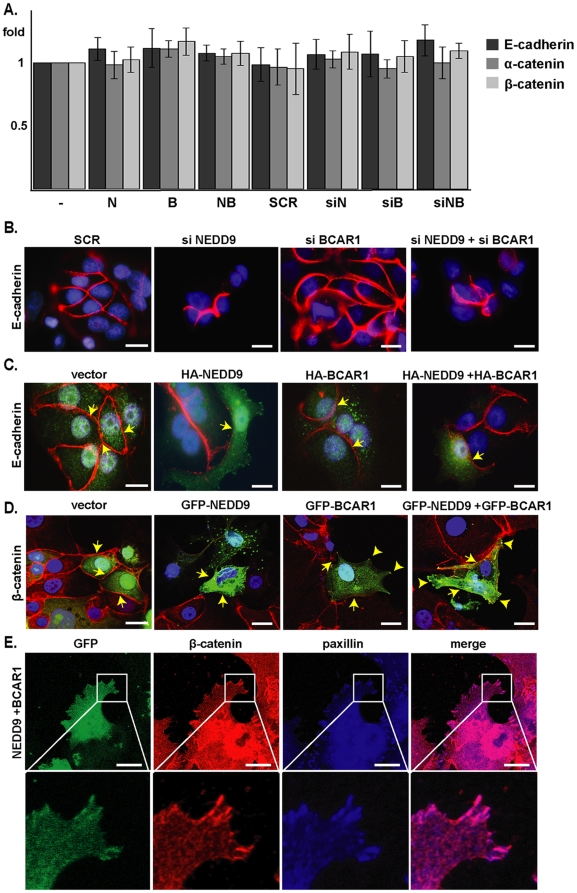
Cas proteins negatively regulate association of E-cadherin with cell junctions. **A**. qRT-PCR of mRNA isolated from MCF7 cells transfected with plasmids including vector pcDNA-HA (-), pcDNA-HA-NEDD9 (N), pcDNA-HA-BCAR1 (B), or pcDNA-HA-NEDD9 and pcDNA-HA-BCAR1 (NB). **B**. MCF7 transfected with siRNAs including scrambled control (SCR), or targeting NEDD9 (siN), BCAR1 (siB) and both (siNB), were stained for E-cadherin (red), and DAPI (blue). **C**. MCF7 cells transfected as in **A** and stained for E-cadherin (red), HA-fused proteins (green) and DAPI (blue). Arrows indicate cell-cell contacts of transfected cells. **D**. MCF7 cells transfected with plasmids including vector pcDNA-GFP, pcDNA-GFP-NEDD9, pcDNA-GFP-BCAR1, or pcDNA-GFP-NEDD9 and pcDNA-GFP-BCAR1 (green) were stained for β-catenin (red), and DAPI (blue). Arrowheads point to β-catenin localization at the focal adhesions. Scale bar in **B–D**, 20 µm. **E**. Immunofluorescence demonstrating that β-catenin localizes to focal adhesions in MCF7 cells expressing pcDNA-GFP-NEDD9 and pcDNA-GFP-BCAR1 (green). β-catenin (red) and the focal adhesion protein paxillin (blue) are indicated. Scale bar, 20 µm. Bottom panel represents magnifications of indicated areas from boxes.

E-cadherin protein expression might be negatively regulated by Cas proteins via several possible mechanisms, including transcriptional downregulation of E-cadherin expression. However, quantitative RT-PCR analysis did not indicate any NEDD9- or BCAR1-dependent changes in E-cadherin mRNA expression (Figure 2A).

Alternatively, NEDD9 and BCAR1 may control association of E-cadherin with the cell surface, which is associated with its rate of degradation. Immunofluorescence analysis of E-cadherin in cells where these proteins were depleted (Figure 2B) showed a thicker band of of E-cadherin at points of cell-cell contact, particularly when NEDD9 was depleted. Reciprocally, overexpression of these proteins in combination resulted in a reduction in the levels of E-cadherin and β-catenin detectable at cell junctions (Figure 2C, 2D). Interestingly, residual β-catenin staining was often detected within intracellular compartments, colocalizing with overexpressed NEDD9 and BCAR1 at focal adhesions (Figure 2D, E).

**Figure 3:**
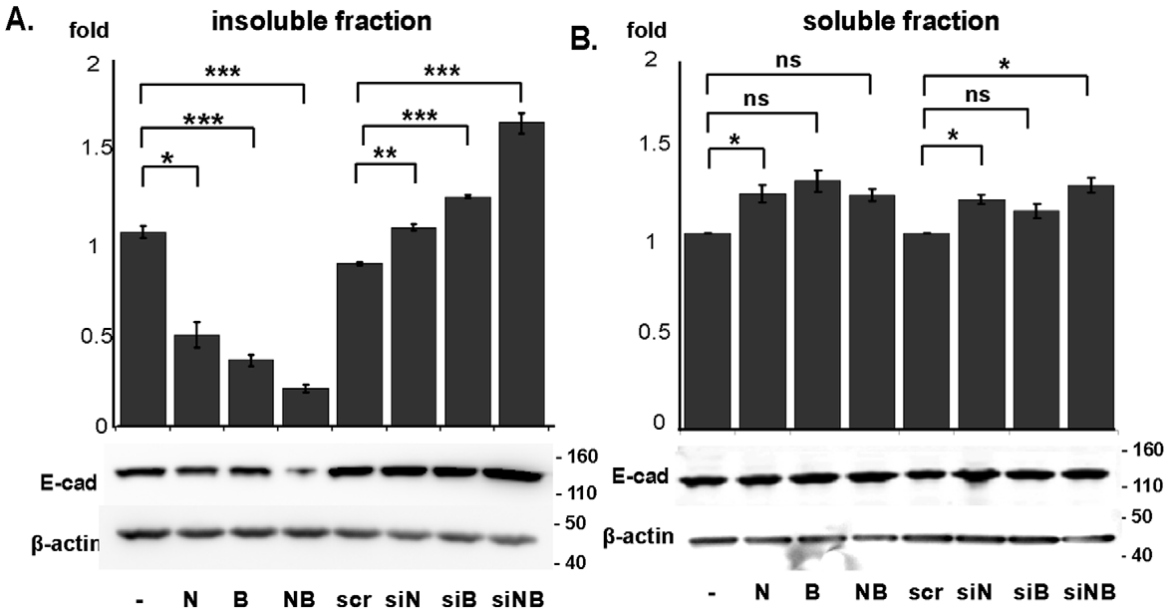
Fractionation indicates Cas proteins decrease E-cadherin association with the insoluble fraction. MCF7 cells were transfected as in **Figure 2A and B**, fractionated to insoluble (**A**) and soluble (**B**) fractions, and then analyzed for expression of E-cadherin. Graphs represent E-cadherin normalized to β-actin in each fraction calculated from 3 independent experiments. Immunoblot represents a typical experiment. *, P<0.05, **, P<0.01, ***, P<0.001. Error bars represent SE.

Subsequent cell fractionation analyses confirmed a specific action of Cas proteins in negatively regulating the membrane- and cytoskeleton-associated, insoluble pool of E-cadherin (Figure 3A). E-cadherin is downregulated from the insoluble fraction in cells overexpressing Cas proteins (2.5-fold in cells with both NEDD9 and BCAR1 depleted), and upregulated in Cas-depleted cultures (2-fold in cells with both NEDD9 and BCAR1 overexpressed). By contrast, the effect of increasing or decreasing Cas protein expression on the levels of the soluble pool of cytoplasmic E-cadherin did not rise to statistical significance (Figure 3B).

### Cas proteins promote lysosomal degradation of E-cadherin via SRC kinase

**Figure 4:**
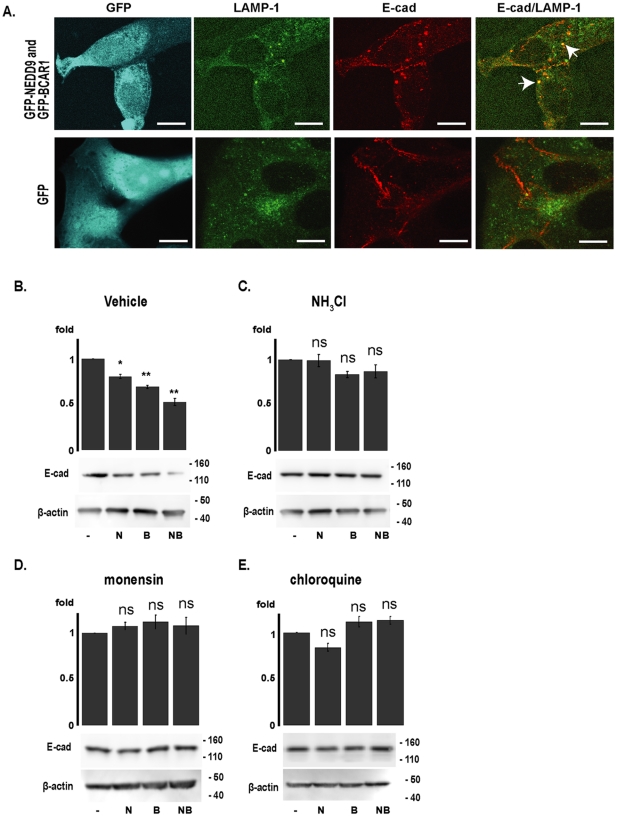
Cas proteins increase lysosomal colocalization of E-cadherin. **A**. Immunofluorescence assessing E-cadherin localization, with lysosomes visualized with LAMP-1 (green) in cells overexpressing GFP-BCAR1 (B) and GFP-NEDD9 (N), indicated in blue, after treatment with cloroquine. E-cadherin is shown in red. Scale bar, 20 µm. Arrows indicate colocalized E-cadherin and LAMP-1. **B–E**. Quantification of western analysis and representative results of whole cell lysates prepared from MCF-7 cells transfected with either the vector pcDNA-GFP, pcDNA-HA-NEDD9 (N), pcDNA-HA-BCAR1 (B), or pcDNA-HA-NEDD9 and pcDNA-HA-BCAR1 (NB), and treated with **B**. vehicle (DMSO), **C**. ammonium chloride (NH_3_Cl), **D**. monensin or **E**. chloroquine, to inhibit lysosomal action. Graphs represents total levels of E-cadherin normalized to β-actin, calculated from more than 4 independent experiments. P values were calculated to compare levels of E-cadherin in vector-transfected cells versus cells transfected with NEDD9, BCAR1 or both within each drug treatment group; *, P<0.05, **, P<0.01, n.s., non-significant. Error bars represent SE.

Upon endocytosis from the membrane, E-cadherin is either recycled to the membrane or degraded in the lysosome. Ammonium chloride, monensin and chloroquine each block lysosomal activity [35]. Immunofluorescence analysis showed that cells treated with cloroquine and overexpressing GFP-tagged NEDD9 and BCAR1 together accumulated more E-cadherin in vesicles marked with lysosomal marker LAMP-1 than control cells overexpressing GFP alone (Figure 4A). Further, ammonium chloride, monensin and chloroquine each effectively blocked the E-cadherin degradation induced by Cas protein overexpression (Figure 4B–E).

**Figure 5:**
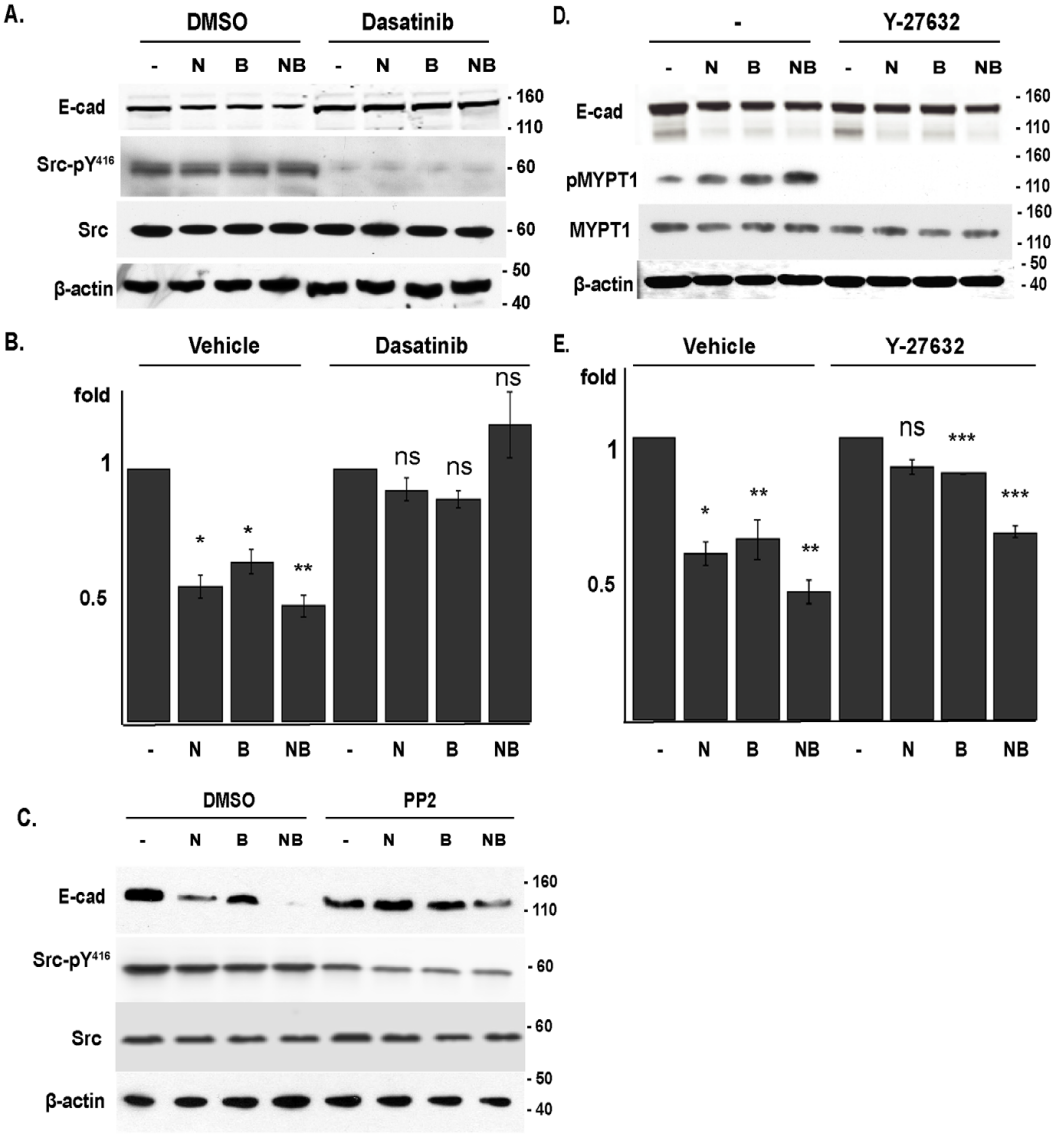
Cas proteins activate SRC to downregulate E-cadherin expression. **A**, **B**. Western analysis (**A**) of whole cell lysates prepared from MCF-7 cells overexpressing pcDNA vector alone (-), or HA-tagged BCAR1 (B), NEDD9 (N), or both (NB), and treated with dasatinib versus vehicle. Quantification of results comparing levels of E-cadherin, normalized to β-actin, within treated and, separately, untreated groups from 4 independent experiments, is also shown (**B**). Src Y^416^ phosphorylation reflects activity state of kinase. P values reflect the difference between the vehicle and drug treatment condition, for each transfected protein indicated; *, P<0.01, **, P<0.001, ns, non-significant. **C**. Experiment as in **A** performed with PP2. Note, SRC inhibition was not as complete as in **A** in these experiments, because at higher PP2 concentrations extensive cell death was observed, probably due to the broader spectrum of PP2 versus dasatinib targets. **D**, **E**. Experiment as in **A**, **B**, but in cells treated with Y27632 versus vehicle. Phosphorylation of the downstream p160ROCK target MYPT1 was assessed in parallel to confirm complete inhibition of p160ROCK (not shown). *, P<0.001, **, P<0.01 ***, P<0.0001, ns, non-significant. Error bars represent SE.

**Figure 6:**
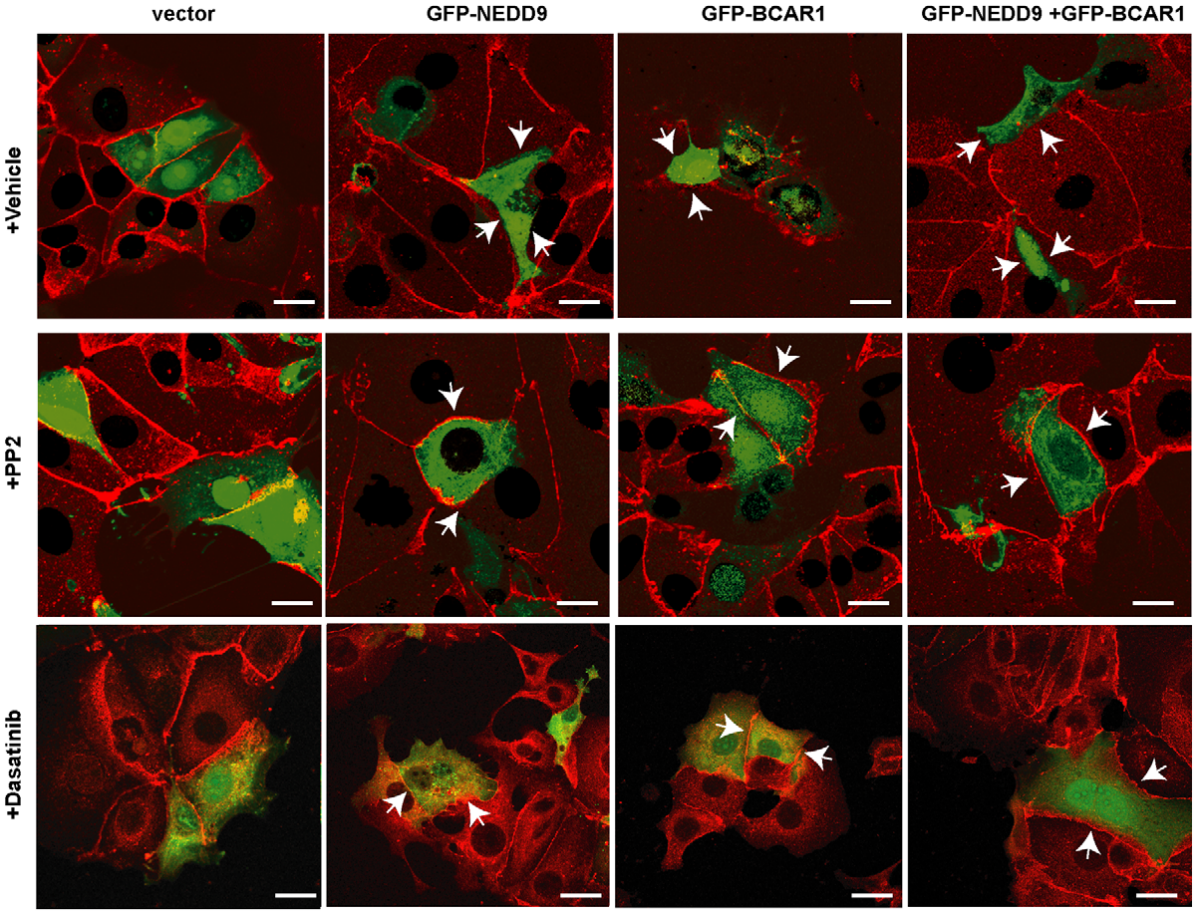
Treatment with SRC inhibitors reverses CAS-induced downregulation of E-cadherin. Immunofluorescence of MCF7 cells expressing vector pcDNA-GFP, pcDNA-GFP-NEDD9, pcDNA-GFP-BCAR1, or pcDNA-GFP-NEDD9 and pcDNA-GFP-BCAR1 (transfected cells shown in green) and treated with vehicle or PP2. Arrows indicate presence of E-cadherin (shown in red) at cell junctions in dasatinib or PP2-treated cells expressing vector, BCAR1 and/or NEDD9, but absence of E-cadherin in similarly transfected cells treated with vehicle. Scale bar, 20 µm.

Activation of SRC causes internalization and lysosomal degradation of E-cadherin [36], [37], [38], while Rho GTPases destabilize adherens junctions by regulating actin polymerization [39] or redistributing E-cadherin on the plasma membrane [40]. Both SRC and RhoA have been directly or functionally linked to Cas proteins [32], [41]. Using dasatinib or PP2 to inhibit SRC kinase (Figures 5A–C), we established that inhibition of Src completely blocked Cas-dependent E-cadherin degradation. In contrast, inhibition of RhoA using Y-27632, a cell-permeable inhibitor of the RhoA effector p160ROCK (Figure 5D, E), had a more limited effect, which did not reach statistical significance if both NEDD9 and BCAR1 were overexpressed. We also found that in addition to reversing loss of the insoluble pools of E-cadherin, treatment with Src inhibitors restored E-cadherin to cell junctions in Cas-overexpressing cells (Figure 6). Together, these results imply that Cas activation of SRC signaling is most important for E-cadherin internalization and degradation.

### MMTV-PyVT tumors arising in a *Nedd9^−/−^* background have increased E-cadherin at cell junctions

**Figure 7:**
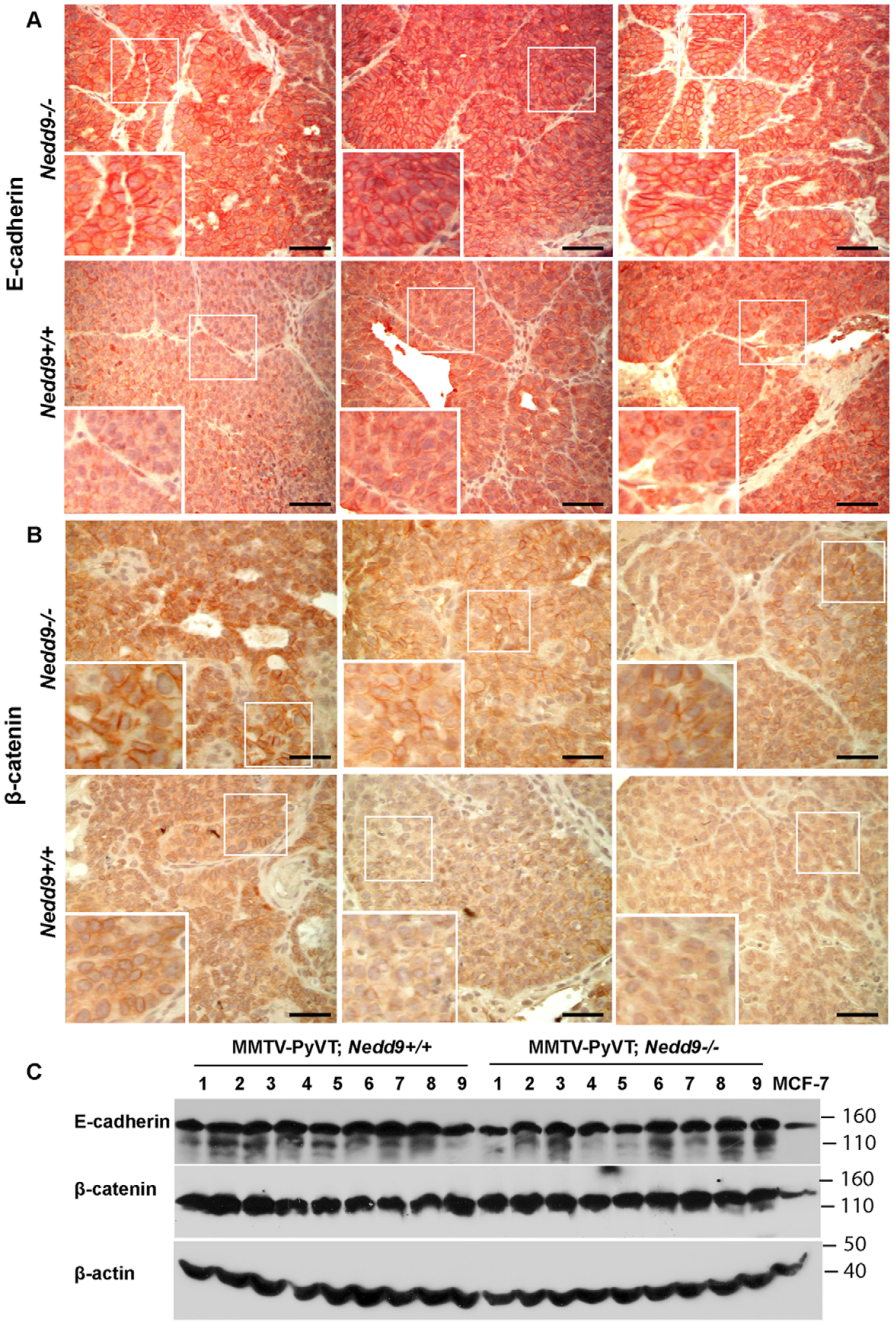
Nedd9*^−/−^* mammary tumors have increased E-cadherin at cell junctions. **A**, **B**. Immunohistochemical evaluation of MMTV-PyVT;*Nedd9^−/−^* and MMTV-PyVT *Nedd9^+/+^* mammary tumors for E-cadherin (**A**) and β-catenin (**B**) protein expression and localization. Insets (thick lines) are magnifications of indicated areas (thin lines). Three representative independently arising tumors are shown for each genotype. Scale bar, 100 µm. **C**. Western analysis of total lysates isolated from 9 independent MMTV-PyVT;*Nedd9^−/−^* and MMTV-PyVT *Nedd9^+/+^* mammary tumors and from MCF7 cells were probed for content of E-cadherin and β-catenin. β-actin served as loading control.

We have reported a longer latency until appearance of mammary tumors in MMTV-PyVT mice, and depressed activation of SRC, in the context of a *Nedd9^−/−^* versus a *Nedd9^+/+^* genotype [22]. We find that loss of Nedd9 protein also caused a noticeable change in the localization of E-cadherin in tumors, with a much more intensive junctional staining pattern consistently observed in *Nedd9^−/−^* tumors, versus a more cytoplasmic distribution in *Nedd9^+/+^* tumors (Figure 7A). A similar result was observed with β-catenin, although response was more heterogeneous (Figure 7B). By contrast, total levels of E-cadherin and β-catenin in tumors were not grossly affected based on Nedd9 genotype (Figure 7C).

## Discussion

This work for the first time establishes that Cas-dependent signaling through SRC kinase promotes E-cadherin removal from cell junctions and its lysosomal degradation in mammalian cells, and suggests this relationship is retained in mammary tumors. Surprisingly, this result differs from our findings in Drosophila, where loss of Dcas increased the total levels of E-cadherin protein in embryos and larvae [34], but the E-cadherin protein failed to localize to the membrane. There are several plausible reasons for the different findings. First, the evolutionary distance between Drosophila and mammals may have resulted in different signaling relationships between Cas proteins and E-cadherin. Second, embryogenesis in the sustained absence of Dcas may lead to the induction of compensatory mechanisms to upregulate levels of E-cadherin in response to lost function at cell-cell contacts. Supporting this latter idea, we observed that in mammals, the inverse relationship between Cas expression and total levels of E-cadherin was more notable following transient manipulations of Cas proteins (Figure 1), rather than in the context of the sustained loss of Nedd9 in mammary tumor development (Figure 7), although the failure of E-cadherin to associate with cell junctions and the detergent insoluble (membrane- and cytoskeleton-enriched) cell fraction was observed in all cases. Third, in Drosophila, the greatest effect on E-cadherin expression was observed when both Dcas and its interacting partner FAK were absent, while this study only examined inhibition of Cas proteins. It is possible that dual inhibition of FAK and Dcas might yield a qualitatively different result regarding E-cadherin expression. However, we view this as unlikely, as we already have reported that FAK activation levels are significantly depressed in *Nedd9^−/−^* versus *Nedd9^+/+^* tumors [22].

Intriguingly, our data show that in cells overexpressing Cas proteins, part of the intracellular pool of β-catenin colocalized with the Cas proteins at focal adhesions (Figure 2). A prior report found direct interactions between β-catenin and the focal adhesion-associated protein paxillin was induced under some cell growth conditions, such as response to injury [42]. It is possible that NEDD9 and BCAR1 overexpression provides similar stimuli, indirectly affecting β-catenin recruitment. Alternatively, an NMR spectroscopic study of BCAR1 identified a domain within this protein that formed a four-helix bundle similar to that found in other proteins such as α-catenin, involved in protein-protein interactions [43], that might serve to directly recruit β-catenin to an anomalous location. At present, the functional significance of this relocalization is unknown in the context of Cas-associated tumorigenesis; this topic requires further study.

It is interesting that NEDD9 and BCAR1 regulation of E-cadherin are not equally inhibited by Y-27632, an inhibitor of p160ROCK. We have previously shown that NEDD9 interacts with the Rho-GEF ECT2 to regulate Rho and p160ROCK activity during mitosis [32]. Reciprocally, Ando et al have found that inhibition of RhoA GTPases and p160ROCK influences the localization and phosphorylation of NEDD9 [44]. No such direct connections have as yet been identified for BCAR1, raising the possibility that NEDD9 may be more active than BCAR1 in its ability to signal through a Rho-p160ROCK effector interaction in interphase, as in mitotic cells.

This study is in general agreement with the recent work by Bui et al. that showed a role for NEDD9 in mediating dioxin-induced EMT [28]. In that study, knockdown of NEDD9 restored a dioxin-induced reduction in E-cadherin expression, while dioxin treatment strongly induced the expression of NEDD9. Although a role for SRC was not examined in that work, SRC has been shown by others to be similarly induced by dioxin, and to directly bind to the aryl hydrocarbon receptor through which dioxin acts [45], suggesting a similar response module is involved in a very different model for tumor cell invasiveness. Taken in sum, these data support the idea that the upregulation of Cas proteins in a subset of tumors promotes tissue invasion in part by removing E-cadherin from the cell surface, and hence disrupting cell-cell junctions.

## Materials and Methods

### Transfection and RT-PCR

MCF-7 breast adenocarcinoma cells were transfected with 20 nM non-targeting scrambled siRNA, or siRNAs to NEDD9 (Hs_NEDD9_2), BCAR1 (Hs_BCAR1_6), or both (Qiagen, Valencia, CA), with RNAiMAX transfection reagent (Invitrogen, Carlsbad, CA). pcDNA3.1-based plasmids containing HA- or GFP fused NEDD9 or BCAR1 were nucleofected into MCF-7 cells using Amaxa V kit (Amaxa, Walkersville, MD). siRNA or plasmid sequences are available on request. mRNA was isolated using RNeasy Mini Kit (Quigen, Valencia, CA). Real-time RT-PCR was performed by the Fox Chase Cancer Center Genomics Facility, using approaches previously described [7]. The probes and primers for E-cadherin and the catenins are available upon request.

### Fractionation and western analysis

For fractionation, cells were lysed in CSK buffer [35] containing 0.5% Triton-X100, incubated 15 min on ice, harvested by scraping and passed 4 times through a 26.5G needle, and then centrifuged for 1 hour at 14,000 g at 4°C. Pellets were solubilized in 3X Laemmli sample buffer containing 10% SDS, then boiled for 20 min. Whole cell lysates were prepared by scaping cells off plates in 3X Laemmli sample buffer containing 10% SDS, and boiling for 20 minutes. Solubilized pellets prepared from 10^5^ cells, and whole cell lysate equivalent to 5×10^5^ cells were used for analysis. Samples were run on 10% Bis-Tris NuPage PAGE (Invitrogen, Carlsbad, CA). Western blots were performed using standard protocols, and signals visualized either by film or by Odyssey (LICOR, Lincoln, NE). Signals in the linear range obtained from western blots were quantitated with Odyssey software, or alternatively with ImageJ software. Significance of data was analyzed by a two-tailed paired-sample t-test in MS Excel.

For drug inhibition experiments, cells were cultured in medium with 1 µM Y-27632 (Calbiochem, San Diego, CA), 25 nM dasatinib (Bristol-Myers Squibb, New York, NY), or 20 nM PP2 (Calbiochem, San Diego, CA) 24 hours post-transfection, and cells were harvested 24 hours later for western blot analysis and RhoA activation assay, using a kit from Cytoskeleton, Inc. (Denver, CO). Inhibition of lysosome activity was achieved by treatment of cells for 24 hours prior to analysis with 2 mM ammonium chloride (Sigma-Aldrich, St. Louis, MO), 10 µM monensin (Calbiochem, San Diego, CA), or 50 µM chloroquine (Sigma-Aldrich, St. Louis, MO).

### Immunofluorescence

MCF7 cells grown on cover slips were washed twice with PBS, incubated in 4% PFA for 20 min, rinsed with PBS and permeabilized in 0.1% Triton X-100 solution for 20 min at room temperature. Samples were blocked with 1% BSA for 1 hour and incubated with primary antibody for 2 hours. Samples were washed and incubated for 1 hour with secondary antibody conjugated with Alexafluor 488, 568 or 633 (Molecular Probes Inc., Eugene, OR) diluted at 1∶1000. Cover slips were mounted onto glass slides in ProLong Gold antifade reagent containing nuclear stain (Invitrogen, Carlsbad, CA) and visualized using a Nikon C1 confocal microscope. Images were analyzed using Metamorph software (Molecular Devices, Sunnyvale, CA). Primary antibodies used included mouse anti-E-cadherin ECCD-2 (Invitrogen, Carlsbad, CA), mouse anti-E-cadherin, rabbit anti-β-catenin ab6302 (Abcam, Cambridge, MA), and anti-β-actin ab6276 (HRP conjugated, Abcam, Cambridge, MA), or anti-β-actin (clone AC-15, Sigma-Aldrich, St. Louis, MO). Secondary antibodies included HRP-conjugated anti-mouse or -rabbit (Amersham, Pittsburgh, PA), IRDye800 or 700-conjugated α-mouse, -rat or –rabbit (LICOR, Lincoln, NE). Low to moderate intensity images were used for quantification of assays, to ensure linearity of signal in immunoblots related to measurement of levels of E-cadherin and β-actin.

### Immunohistochemistry

The derivation and preparation of the MMTV-PyVT mammary tissues for immunohistochemistry has been described in detail [22]. Briefly, a standard two-stage indirect immunoperoxidase staining protocol was used for all tissues (Histostain-Plus Kit, Invitrogen, Carlsbad, CA). Citrate based antigen retrieval buffer was from BD Biosciences, San Jose, CA). As controls, sections were stained with a control rabbit IgG or diluent alone (5% goat serum in Tris-buffered saline). All tissue sections were incubated at room temperature for 1 h with primary antibodies and 30 minutes with secondary antibody solution; these and subsequent procedures were performed according to the instructions provided with the kit. Sections were counterstained with hematoxylin (Sigma, St Louis, MO). Rabbit antibodies to E-cadherin (ab53033) and β-catenin (ab6302) from Abcam (Cambridge, MA) were used at a dilution of 1∶100. Images were acquired at 40x with a Nikon Eclipse E600 microscope.
